# Chronically ill patients’ perspectives on support services and activities of patient organizations

**DOI:** 10.1186/s13584-024-00635-7

**Published:** 2024-09-16

**Authors:** Avi Zigdon, Eyal Eckhaus, Michal Rosenfeld, Ofek Zigdon

**Affiliations:** 1 Department of Health Systems Management, School of Health Sciences, Ariel University, Science Park, P.O.B. 3, Ariel, 40700 Israel; 2 Ramat Gan Academic College, Pinchas Rotenberg 87, Ramat Gan, 52275 Israel; 3 Maccabi Healthcare Services, Tel-Aviv, Israel; 4 Faculty of Medicine, Hebrew University of Jerusalem, Ein Kerem., P.O.B. 12271, Jerusalem, 9112102 Israel

**Keywords:** Patient Organizations (POs), Patient perspectives, Chronically ill patients, Patient-oriented questionnaire, Services and activities, Health policy, Healthcare system

## Abstract

**Background:**

Patient Organizations (POs) are an important support factor in helping chronically ill patients cope with their illness. Patient involvement in the management of their disease helps to achieve the best possible care for the patient, streamline the work of healthcare providers, shape healthcare policy, and even influence the structures of healthcare systems. The perspective of chronically ill patients on the activities and services provided by patient organizations has not been evaluated yet. This study aimed to identify and map the services and activities of all types of non-profit patient organizations from the perspective of chronically ill patients so that they can be integrated as an integral part of the healthcare system.

**Methods:**

Nineteen services and activities of patient organizations were sampled from Israeli patient organizations and scientific literature. These services and activities were evaluated by chronically ill patients in Israel. Patient-Oriented Questionnaires (POQ) were distributed among patients with chronic diseases (*N* = 1395) using snowball sampling.

**Results:**

Exploratory factor analysis (EFA) was performed, followed by confirmatory factor analysis (CFA) for convergent and discriminant validity. Findings showed that twelve services and activities suggested by patient organizations were found to represent chronically ill patients’ needs and categorized into three groups: Interpersonal support (five items), patients’ rights (four items), and medical information (three items). CFA showed a good fit for the observed data. CFI = 0.98, NFI = 0.97, TLI = 0.96, RMSEA = 0.058.

**Conclusions:**

Well-organized patient organizations are an important pillar in reformed healthcare systems. They can serve as the social arm of the healthcare system and as an intermediary between patients and healthcare institutions. We narrowed down twelve services and activities given by patient organizations that were important to chronically ill patients in Israel. patient organizations can utilize patient needs or preferences into clinical practice and influence health policy planning, patient-caregiver relationships, research and even healthcare costs. patient organizations recognition by the healthcare system, and establishment of a national patient council will help to realize these processes.

**Supplementary Information:**

The online version contains supplementary material available at 10.1186/s13584-024-00635-7.

## Introduction

Global morbidity and mortality are mainly caused by chronic diseases [1]. Patients diagnosed with a chronic disease feel disappointed with the quality of care, lack of availability of human resources, poor access to information, and inadequate responsiveness in the healthcare system [2, 3]. They are interested in assuming a greater role in the management of their treatments [4]. Strategies that encourage patient involvement in managing their illness help to achieve the best possible care for the patient, streamline the work of healthcare providers, and shape health policies [5, 6]. Effective communication and trust between patient and caregiver reduce health disparities and promotes health equality [7], and there is even evidence of the impact of patients’ perceptions on the structures of healthcare systems [8, 9].

In recent decades, new conceptions of the patient’s role in the therapeutic process have emerged [10]. These believe that it is of great importance for the patient to be placed at the center of the treatment process. The patient must be provided with all the tools and information needed to maintain active involvement in the decision-making process [10]. Patients occupy an important place in providing emotional support to other patients. But in recent years they have been offering other patients’ guidance in the field of personal health, based on the experience they have acquired from managing similar health conditions, and the patient’s experience has been defined as experiential knowledge acquired personally from the day-to-day management of the disease. The support offered by patients to other patients differs from the support offered by their physicians in type, style and topic, due to the different experience in managing the disease [11]. Patients or family members who have gained experience in dealing with their illness often organize themselves into patient organizations. This allows them to share the knowledge they have acquired with other patients at the beginning of their treatment journey.

These organizations play an important role in supporting chronically ill patients. They are the connecting threads that facilitate communication of information between patients and health care providers. They provide telephone counseling, offer online social network support [12], provide medical rights information [6], and specialize in achieving the desires and needs of the patients [13, 14]. They serve as an administrative link. They are funded by the government and healthcare system [4]. They work to empower patients [6] and improve their health [15]. These organizations play an influential political role in shaping health policies. They assist in recruiting patients to accelerate research, financially supporting both patients and research programs [16–22]. They work to share evidence-based medical knowledge, experiences, and preferences in managing a particular disease [23]. In most organizations, services and activities are provided at little to no cost and some even serve as social support groups protecting the patient from the disease’s negative effects on the quality of life [24]. Some patient organizations have been set up by patients who share the same experiences and provide emotional and practical support from their own experiences [12, 25]. Namely, Patient Advocacy Organizations (PAO) [15]; Patient Advocacy Groups (PAG) [26]; Support Groups (SG) [24]; Patient Organizations (PO) [6] and Group Education (GE) [23].

Research shows that patients who are members of patient organizations rate their health better, are more satisfied with their treatment, and are more knowledgeable about techniques and treatment innovation than people who are not members of patient organizations [27, 28]. However, some patient organizations policies limit their capability of providing an ideal personalized plan of care for each patient as a result of their financial dependency on the pharmaceutical industry and medical device manufacturers [6, 29]. The number of patient organizations has increased in recent years, and they compete for funding and visibility [30]. To our knowledge, services and activities of patient organizations have yet to be studied from the patient’s perspective, notably the services, that assist the patient with managing their disease. The aims of this preliminary study were to identify and map the services and activities of all types of non-profit patient organizations from the general chronically ill patient’s perspective so that they can be integrated as an integral part of the healthcare system.

## Materials & methods

### Study design

A prospective study was conducted among chronically ill patients in Israel, to identify and map the services and activities suggested by patient organizations from the chronically ill patients’ perspective. The participants were sampled using snowball sampling by pre-instructed research assistants. All research participants were aged ≥ 18 years. Non-lucid participants or those with cognitive impairments were excluded from this study. The questionnaire was developed using the Qualtrics system.

The research questionnaire was distributed in two phases. In the first phase, questionnaires were independently filled out by the participants after signing an informed consent form. Each participant who reported having a chronic illness, either in the past or present, was asked to refer additional individuals who, to the best of their knowledge, also had a chronic illness. This referral formed the second phase of the sampled population.

To estimate the required sample size, we relied on data from the Central Bureau of Statistics in Israel. As of January 2024, the population of the State of Israel was estimated to be 9.855 million [31]. The proportion of chronically ill patients in the population, who reported to have one or more chronic illnesses, was estimated to be 21% [32], amounting to approximately 2.07 million people. Although this figure includes children up to the age of 18, and the specific population of chronically ill patients between the ages of 0 and 18 years is not detailed, it was decided to use this population figure (*N* = 2,070,000) to calculate the sample size.

At a confidence level of 95%, considering the unpredictable participation rate of patients in the activities and services of patient organizations, a rate of 50% was used to obtain the largest minimal sample size. Using these parameters, a minimal sample size of 384 patients was calculated for this study. Since the aim of this study is to identify broad trends among the general chronically ill patient population in Israel, the sample wasn’t categorized to specific illness type, nor by socio-demographic or geographic parameters.

### Ethical considerations

Ariel University’s research ethics committee reviewed and approved all experimental protocols (ref AU-AZ-20180307). Written informed consent was obtained from all the participants prior to completing the questionnaire. Questionnaires were coded for anonymous data analysis. The type of disease and health status were self-reported by the participants.

### Participants

The total amount of filled-in questionnaires was 1,876, of which 481 were excluded either because they were incomplete and missing data could not be obtained or the participant did not report a chronic illness.

Of the valid 1,395 completed questionnaires, 842 (60.4%) were female participants and 553 (39.6%) were male participants. Furthermore, 23.7% were between the ages of 37–55 (*n* = 125 males; *n* = 206 females), 23.5% between the ages of 56–70 (*n* = 119 males; *n* = 209 females), and 52.8% were ≥ 71 years old (*n* = 309 males; *n* = 427 females). Additionally, data on gender, marital status, country of birth, nationality, educational level, reported health status, and type of disease were collected. Respondents’ characteristics are summarized in Table 1.

**Table 1:** Respondents’ characteristics (*N* = 1395)

	Respondents, *n* (%)
Gender	
Female	842 (60.4)
Male	553 (39.6)
Marital Status	
Married/Partner	891 (63.9)
Never Married	233 (16.7)
Divorced	147 (10.5)
Widowed	117 (8.4)
Missing	7 (0.5)
Country of Birth	
Israel	741 (53.1)
Other	654 (46.9)
Religion	
Jewish	990 (71)
Muslim	292 (20.9)
Christian	78 (5.6)
Druze	11 (0.8)
Other	24 (1.7)
Education level	
University	555 (39.8)
College	253 (18.1)
High School or below	566 (40.6)
Missing	21 (1.5)
Reported Health Status	
Very Good	205 (14.7)
Good	636 (45.6)
Not so good	377 (27)
Not good	114 (8.2)
Bad	56 (4)
Chronic disease	
Diabetes	397 (28.5)
Cardiovascular disease	206 (14.8)
Asthma or lung disease	161 (11.5)
Cancer	108 (7.7)
Arthritis	105 (7.5)
Mental Disorders	103 (7.4)
Osteoporosis	49 (3.5)
Stroke (CVA)	44 (3.2)
Parkinson’s disease	20 (1.4)
Multiple Sclerosis (MS)	18 (1.3)
Cystic fibrosis	4 (0.3)
Other	180 (12.9)

### Measures

The research questionnaire included three main parts. The first part consisted of two filter questions. The second part consisted of questions about patient organizations and the third part included demographic questions. The first filter question was: “Do you suffer from any disease?” If yes, the second question was: “What is the disease you are suffering from?”. The questionnaire was administered if the self-reported disease was considered chronic. The second part examined the participant’s familiarity with patient organizations, the services, and activities they provide: “Do you know if there is a patient organization for your disease?“. The next question regarded the perceived usefulness of services and activities supplied by patient organizations: “The purpose of patient organizations is to help patients through services and activities that they initiate, you will be presented with services and activities that can be obtained from various patient organizations. Please indicate how much the presented service or activity can help you in managing your illness?”

The questionnaire included 19 items that were developed based on the literature [6, 12–14, 16–23] and activities of patient organizations in Israel. The services and activities were presented equally to all participants as shown in Table 2, in the same order in each of the questions, the participant was required to rate his or her answers on a 4-level ordinal scale (1-would not help at all to 4-would be very helpful). The research tool was content-validated by three patient organization managers, two Ph.D.-Level researchers, and a patients’ rights specialist. After slight wording notes, appropriate changes were made into the items and the questionnaire was distributed. To test the reliability of item variables and the quality of variable comprehension, a pilot study was conducted among 40 chronically ill patients. The pilot study found a reliability level with Cronbach’s α = 0.862 for all items and no wording problems were found. The third part included demographic questions: gender, marital status, country of birth, religion, education level, reported health status and self-reported on chronic disease.

**Table 2:** Services and activities suggested by Patient organizations: patient-oriented questionnaire (POQ)

	On a scale from “1-would not help at all to 4-would be very helpful”
Item	Variable
Q1	Attending medical conferences and seminars on the disease
Q2	Receiving information about the disease and treatment
Q3	Online medical information
Q4	24/7 Hotline
Q5	Patient social gatherings
Q6	Personal support meetings with another patient organization member
Q7	Phone consulting with a patient organization professional
Q8	Support group meeting with other patient organization members
Q9	Weekend holiday with other patient organization members and their families
Q10	Online support forum
Q11	Clubs (e.g. Pilates, supporting exercise)
Q12	Patients’ rights information
Q13	Assistance in utilizing patients’ rights
Q14	Patient active involvement in research and new treatment development
Q15	Financial support for treatments
Q16	Professional caregiver (non-patient), trained and familiar with the healthcare system
Q17	Family member, trained and familiar with the healthcare system
Q18	Attend a course where you will learn to become better acquainted with your disease
Q19	Experienced patient, trained and familiar with the healthcare system

### Data analysis

Exploratory Factor Analysis (EFA) was performed in the first half of the data (696 participants), followed by Confirmatory Factor Analysis (CFA) for convergent and discriminant validity [33]. On the other half of the data (699 participants). This splitting technique confirms that the model developed by the EFA is consistent. Model fit was estimated using Comparative Fit Index (CFI), Tucker-Lewis Index (TLI), Normed-Fit Index (NFI), and Root Mean Square Error of Approximation (RMSEA) [34]. Values ​​of CFI, NFI, and TLI ≥ 0.95, and RMSEA ≤ 0.06 are considered a good fit [35]. We used SPSS v.24 for EFA and AMOS v.24 for CFA.

## Results

### Exploratory factor analysis

Kaiser-Meyer-Olkin measure of sampling adequacy was 0.89 which was above the recommended value of 0.6, and Bartlett’s test of sphericity was statistically significant (χ^2^ (105) = 4564.06, *p* < 0.001). A principle-components factor analysis of the 19 items using varimax rotations was then conducted. The literature varies for the minimum loading, ranging between 0.4 and 0.5. Therefore, for a rigorous solution we considered a minimum loading of 0.5. after suppressing loadings below 0.5 there were no cross loadings. Four items had low loadings and therefore suppressed, these were Q4, Q11, Q18, Q14.

Eigen-values showed that each variable loaded onto three factors, explaining 60.45% of the variance. These were: (1) Interpersonal support, (2) Patients’ rights, and (3) Medical information. Given these overall indicators, factor analysis was deemed to be suitable for the 15 items. Factor loadings are displayed in Table 3.

The items removed were:

- Q11- Clubs (e.g. Pilates, supporting exercise).
- Q14 - Patient active involvement in research and new treatment development.
- Q18- Attend a course where you will learn to become better acquainted with your disease.
- Q4 24/7 Hotline.

**Table 3:** Factor loadings based on a principal components’ analysis with varimax rotation

Item #	Interpersonal support	Patients’ rights	Medical information
Q1	0.512		
Q5	0.82		
Q6	0.774		
Q7	0.669		
Q8	0.84		
Q9	0.681		
Q19	0.645		
Q12		0.749	
Q13		0.816	
Q15		0.766	
Q16		0.625	
Q17		0.583	
Q2			0.608
Q3			0.857
Q10			0.713

Note. Factor loadings < 0.5 were suppressed

**Fig. 1:**
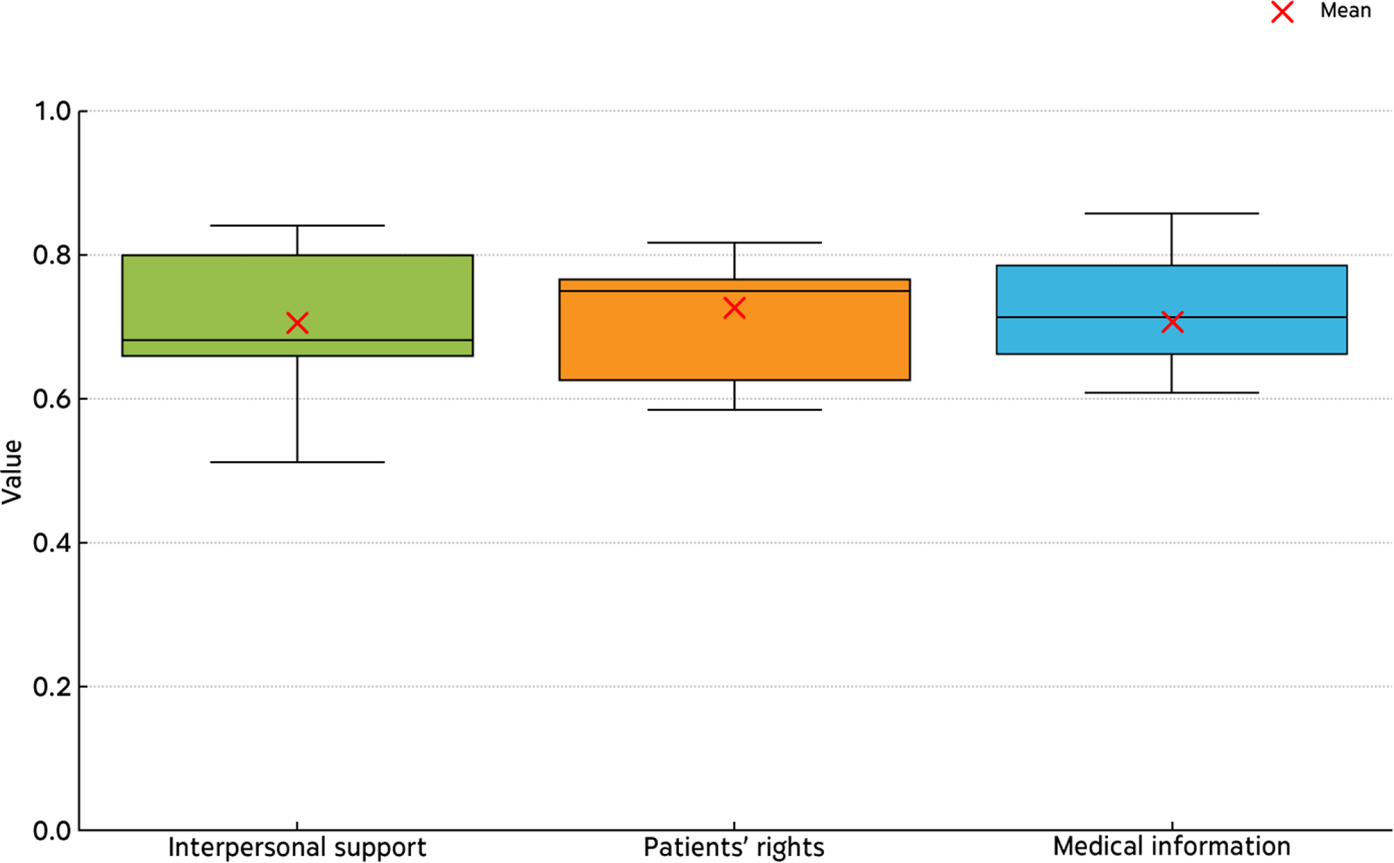
The three constructs’ loadings variation

Cronbach’s α examined reliability, namely internal consistency for the scales. The scales showed adequate alphas of 0.71 for medical information, 0.8 for patients’ rights and 0.87 for interpersonal support. Scale α = 0.88 for the complete scale. The SD of the constructs is: 0.87 for Interpersonal Support, 0.83 for Medical Information, and 0.77 for Patients’ rights. Figure 1 presents the means, variations and skewness of the different latent variables and scales. Boxes represent the interquartile range, and whiskers show the range of the data.

### Confirmatory factor analysis

We used AMOS v.25 for the CFA. Three items (# 1,7,16) were removed to improve model fit. Next, items of each measure were loaded on a specific latent variable: three items for medical information, four items for patients’ rights and five items for interpersonal support.

While ANOVA is typically used to compare nested models, in this case we have latent variable models, so ANOVA is not an appropriate method for comparison. For comparing latent variable models, the chi-square difference test is recommended instead of ANOVA [36].

**Fig. 2:**
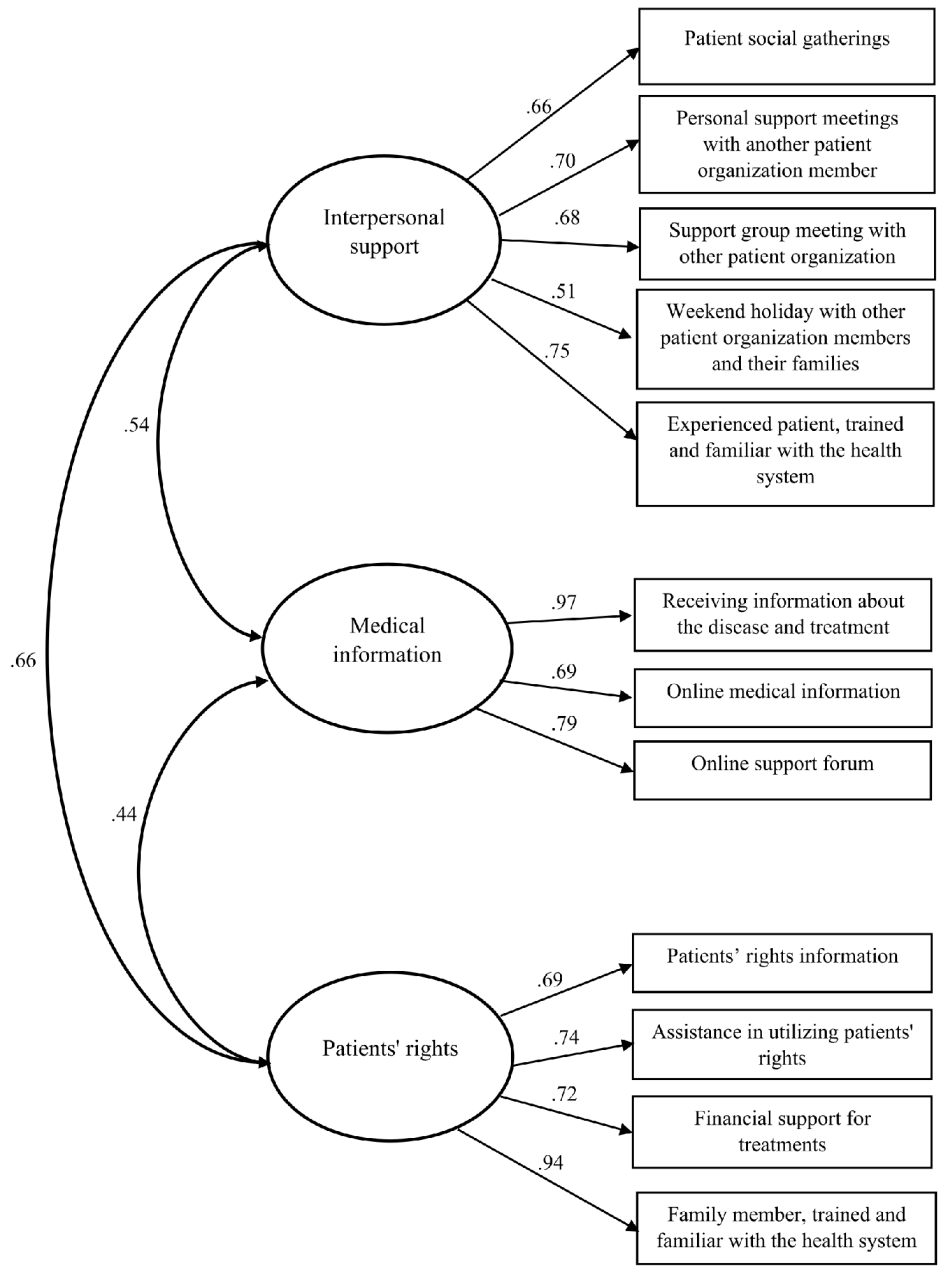
Confirmatory factor analysis of the patient-oriented questionnaire. Fit of the model: CFI = 0.98, NFI = 0.97, TLI = 0.96, RMSEA = 0.058, chi-sqr = 121.6 (df = 36)

Before removing the three items, fit indices were: CFI = 0.95, NFI = 0.94, TLI = 0.91, RMSEA = 0.076, Confidence intervals for RMSEA [0.067, 0.084], Chi-square = 284.18 (df = 57, *p* = 0). After the removal CFA showed a good fit for the observed data. CFI = 0.98, NFI = 0.97, TLI = 0.96, RMSEA = 0.058, Confidence intervals for RMSEA [0.047, 0.070], Chi-square = 121.6 (df = 36, *p* = 0). (Figure. 2 presents the model). In the EFA stage, items are removed because they do not adequately represent or contribute to the underlying latent constructs. The primary purpose of item removal during CFA is to achieve a well-fitting measurement model that accurately represents the hypothesized factor structure and the relationships between the latent constructs and their indicators (items). That is, item removal during EFA is primarily aimed at refining and purifying the factor structure, while item removal during CFA is focused on achieving a well-fitting measurement model that accurately represents the hypothesized factor structure. Both stages contribute to the development of a valid and reliable statistical construct by identifying and retaining the most relevant and representative items.

When comparing with the one factor model, the fit indices for the one factor model did not achieve model fit. CFI = 0.998, NFI = 0.997, TLI = 0.87, RMSEA = 0.1, Confidence intervals for RMSEA [0.043, 0.169], Chi-square = 7.92 (df = 1, *p* = 001). Chi-Square test also depends on the degrees of freedom, models with fewer parameters may show a lower Chi-Square value, even though they display a poorer fit to the data [37]. Therefore, the lower Chi-Square value for the one-factor model (7.92) compared to the three-factor model (121.6) can be explained by the difference in degrees of freedom between the two models- the one-factor model has only 1 degree of freedom, while the three-factor model has 36 degrees of freedom.

Figure 2 Groups of important services and activities given by patient organizations. Every group is supported by the services and activities with the highest loadings in relation to the group. The higher loading values suggest a stronger correlation between the service or activity and the group.

Finally, illness effect on the latent constructs was explored, compared to those who do not suffer from them, by adding illness variables to the model. Table 4 presents standardized regression weights of illness effect on the latent variables.

**Table 4:** Standardized regression weights of illness effect on latent variables

	Interpersonal support	Medical information	Patients’ rights
Diabetes	0.08**	0.068	-0.143*
Cardiovascular disease	-0.038	-0.088	-0.116**
Asthma or lung disease	-0.032	0.005	-0.007
Cancer	0.072**	0.075	-0.026
Arthritis	0.05	0.084	-0.011**
Mental disorders	0.091***	0.049	0.026
Osteoporosis	0.007	0.039	-0.039
Stroke (CVA)	0.057*	0.032	-0.028
Parkinson disease	0.025	-0.014	-0.044
Multiple sclerosis (MS)	0.001	0.034	0.01
Cystic fibrosis	0.016	-0.024	-0.007

*p < .05, **p < .01, ***p < .001

The table above shows that patients dealing with mental depression, cancer, diabetes, or stroke (CVA) were found to have significantly associated with “Interpersonal Support”. Moreover, patients with cardiovascular disease, diabetes or arthritis show a significantly negative associated with “Patients’ Rights”.

## Discussions

Previous studies have not thoroughly examined the services provided by patient organizations from the perspective of those they are intended to support, namely patients coping with chronic diseases. This study examines the contribution of patient organizations’ services and activities from chronically ill patients’ perspectives using a structural comparison approach. Confirmatory factor analysis substantiated the structural integrity of the model proposed in this study and found three main groups as important services provided by patient organizations: “interpersonal support”, “medical information” and “patients’ rights”. “Interpersonal support” is delineated by five elements that address patients’ desire for social contact with like-minded people facing similar health challenges. “Medical information” consists of three main cornerstones: Information about the disease and treatment, online availability (e.g. forum, hotline) and online medical information. “Patients’ rights” focus on four aspects predominantly centered around financial support. These findings underline the needs of patients during their chronic illness.

“Interpersonal support” demonstrates the patients’ need for deep human connections with other patients who share similar health problems. It is based on five services and activities that were considered in the research model: “Patient social gatherings”, “Personal support meetings with another patient organization member”, “Support group meeting with other PO members”, “Weekend holiday with other patient organization members and their families” and “Experienced patient, trained and familiar with the health”. Studies show that patient organization meetings play a protective role against negative disease effects and emphasize the necessity of face-to-face interactions [25]. Chronically ill patients seek to be autonomous in managing their illness [24] and thus would avoid paternalistic dynamics with advisors. Patients expect open communication with patient organization members, allowing for a two-way exchange of information and deliberation about medical recommendations [23]. These processes are well illustrated by staff member of the patient organization SCRC (Shanghai Cancer Recovery Club) in China. “’The Western model of conquering disease highly relies on medical science and technology, but we Chinese patients succeed because of our collective organic social interactions” [38]. Q7 (Phone consulting with a patient organization professional), which was excluded, supports the notion that a patient organization-assigned advisor may be less desirable because it resembles a physician-patient relationship (which is a paternalistic-natured relationship). In formal medical settings, patients often struggle to express misunderstandings about their care, which can jeopardize medical care [39]. It can therefore be assumed that certain procedures in the treatment of the chronically ill can be communicated to patients by patient organizations, thus positively influencing adherence to treatment and allows healthcare providers to concentrate on critical clinical aspects of care.

The findings indicate that chronically ill patients prioritize receiving medical information from their patient organizations. “Medical information” is another issue of concern to patients in connection with patient organizations, as indicated by the activities and services: “Receiving information about the disease and treatment”, “Online medical information” and “Online support forum”. This finding aligns with previous research showing that sharing medical information is very important patient organizations’ activities [14]. Receiving medical information from patient organizations is often done online and may not be always evidence-based. While social media platforms are instrumental for gathering medical information and addressing patient needs, they may not always provide reliable information [40]. However, patient organizations’ websites provide insufficient medical information, and the methods of communicating medical information need to be improved to enhance communication between patient organizations and patients [41]. The evidence-based medical information that can be found online is difficult and cumbersome to understand and raises anxiety among patients [23]. In this study, we found that chronically ill patients notably prefer to receive informal medical information face-to-face from others experiencing the same condition. This provides them with a deep human connection while receiving medical information [12, 25]. The institutionalization and development of control mechanisms by healthcare system representatives over the medical information conveyed to patients by patient organizations and the integration of specialist physicians into the board of patient organizations will make it possible to monitor the reliability of the medical information given to patients, this can potentially improve patients’ clinical outcomes [14]. Support for that can be found in the exclusion of Q1 (Attending medical conferences and seminars on the disease) from the model, which represents receiving evidence-based information about the disease in a public manner.

The third outcome highlighted by the model pertains to patients’ rights, which incorporates four crucial services: “Patients’ rights information”, “Assistance in utilizing patients’ rights”, “Financial support for treatments” and incorporating “Family member, trained and familiar with the healthcare system”. The issue of patients’ rights is well-established, with previous studies supporting this finding [6, 14]. Particularly in Israel, the emphasis on patients’ rights often centers around financial aspects such as allowances, medication costs, medical devices, income tax exemptions, and discounts [42]. Therefore, it can be inferred that the “patients’ rights” group predominantly focused on the financial aspects of patient organizations services. However, the involvement of a “family member, trained and familiar with the healthcare system” introduces a new insight that enriches the existing literature.

A reasonable concern may arise that the state might withdraw some of its responsibilities in response to the high resilience and effectiveness of patient organizations. However, historical data and professional literature suggest that social initiatives have been adopted by government organizations and have become integral parts of government systems. For example in Israel, cancer prevention and early detection programs began as private initiatives led by civil society organizations, including the Israel Cancer Association (The national program for early detection of breast cancer in 1995, and national program for early detection of colorectal cancer in 2005) [43, 44], and the Israeli Lung Cancer Foundation, whose pilot program for the early detection of lung cancer using low-dose CT scans was adopted by the Ministry of Health in Israel in 2021 [45]. Nevertheless, it often took several years before these prevention programs, initiated by social organizations, were embraced as national programs under the Ministry of Health’s supervision and control. Further supporting this integration, a policy document produced by the Prime Minister’s Office in Israel in 2008 included the following in the Prime Minister’s opening remarks: “Some view civil society organizations as a threat that must be defended against; however, they are mistaken. In our view, even the most critical voices within these organizations are partners whose contribution to Israeli democracy and the country’s value strength is as important as anything else.” [46]. Similarly, around the world, the involvement of patient organizations is designed to improve both equality and efficiency [47], and they face challenges in balancing democratic representation, reflecting the key role of patient organizations in the healthcare system [48]. Despite this, socio-economic disparities can influence patients’ health outcomes [47]. Even though integrating new initiatives within the healthcare system may present difficulties, the involvement of patient organizations alongside consistent monitoring and support of the healthcare system, can significantly benefit patients by improving their health literacy, enhancing patient equality and involvement, and potentially reducing the overall burden on the healthcare system.

The health literacy of family members who are patient-oriented can significantly contribute to a patient-centered approach in healthcare. It is essential for patients and their families to be more actively involved in healthcare decision-making and have better access to information and support [49]. The involvement of an informed family member has been shown to play a crucial role in the disease management process, probably due to the high accessibility of the “source of information” regarding patients’ rights to the chronically ill patient. When family support is actively integrated into the activities of patient organizations it can enhance the interface between patient organizations and the healthcare system, particularly in situations where the patient is unable to make medical decisions independently. This integration supports administrative efficiency, which in turn enables patients to better manage their own care, focus on their needs, and receive the necessary support for living with chronic or life-limiting illnesses within the healthcare system [50, 51]. Interestingly, the item “Patient active involvement in research and new treatment development” was excluded, possibly because non-terminal patients or those with multiple treatment options are less likely to participate in clinical trials [52]. This exclusion reflects the existing gaps between the needs of scientific research and the perspectives of patients on research participation [16]. To bridge this gap, Patient organizations could play a vital role in improving and clarifying the understanding of research objectives, processes, and the benefits to the broader public. By doing so, Patient organizations can potentially increase patient participation in research, contributing to the development of new treatments and the advancement of medical knowledge [53].

### Limitations

The current study had some limitations which should be considered. Snowball sampling was not representative of the general chronically ill patient population and did not allow for a specific patient population to be reached. In this respect, researchers had to remove a relatively large group of study participants. The type of chronic illness was self-reported, and no clinical tests took place to confirm the participant was indeed suffering from a chronic illness. This study brought into play a variety of services and activities that patient organizations gave to chronic patients, but it is possible that there were patient organizations that gave additional services and activities that were not revealed in this study. The research population included patients with a variety of chronic diseases that showed a wide spectrum of services and activities. Had the research been conducted on a specific population with a single chronic disease, indeed, different outcomes may have appeared. Using the POQ – Patients Oriented Questionnaires in follow-up studies on patient organizations that provide services and activities for chronically ill patients with the same disease will help focus patient organizations activity, and minimize resources wasted on services and activities that are not needed by chronically ill patients.

## Conclusions

In recent years, there has been an increasing recognition of the importance of involving patients and patient organizations in health care decision-making processes, leading to a rise in the number of countries and organizations working towards this goal. Based on our study conducted in Israel, we identified 12 key services and activities provided by patient organizations that are particularly valuable to chronically ill patients. These services and activities were categorized into three primary groups: medical information, interpersonal support, and patients’ rights. These categories offer a clearer understanding of the expectations chronically ill patients have from patient organizations. For patient organizations to effectively meet the needs of these patients, they should prioritize efforts and resources on disseminating information about diseases and treatments, facilitating personal support meetings, and providing training for family members. By doing so, Patient organizations can enhance resource efficiency to benefit both patients and their families. The use of the Patients Oriented Questionnaires (POQ) can guide patient organizations in delivering services and activities that align with the genuine needs of chronically ill patients, thereby improving patients’ ability to manage their conditions more effectively. Well-organized patient organizations represent a crucial component of reformed healthcare systems. Therefore, we recommend that the Ministry of Health formalize and recognize the unique role of patient organizations within the healthcare system by establishing a national patient council. This council would facilitate the realization of these processes and promote collaboration between patient organizations and healthcare providers and should be composed exclusively of patients and patient representatives to ensure that patients’ interests are represented in the most effective and unbiased manner. Including representatives from the pharmaceutical industry, healthcare providers, health maintenance organization or medical professionals on the patient council could compromise the decision-making process for patients, as these stakeholders may have economic and business interests that do not necessarily align with the sole well-being of the patient.

Patient organizations can function as the social arm of the healthcare system serving as intermediaries between patients and healthcare institutions. They have the potential to integrate patients’ needs and preferences into clinical evidence, influence health policy planning, shape patient-caregiver relationships, contribute to research and even impact healthcare costs.

## Electronic supplementary material

Below is the link to the electronic supplementary material.

Supplementary Material 1

## Data Availability

The data used in this study is available from the authors. However, Ariel University’s research ethics committee’s and the School of Health Sciences’ approvals are required upon reasonable request.

## Abbreviations

PO: Patient Organization
POQ: Patient-Oriented Questionnaire
PAO: Patient Advocacy Organizations
PAG: Patient Advocacy Groups
SG: Support Groups
GE: Group Education
