# Supplementary material for: Chronically ill patients’ perspectives on support services and activities of patient organizations
Source: Isr J Health Policy Res. 2024 Sep 16;13:47. doi: 10.1186/s13584-024-00635-7 (PMC11403880; doi:10.1186/s13584-024-00635-7)
Supplement: Supplementary file 1 — Supplementary Material 1 [file 13584_2024_635_MOESM1_ESM.docx]

Manuscript ID: JHPR-D-23-00131

Israel Journal of Health Policy Research

Dear Prof Avi Israeli, Editor

Thank you for the opportunity to revise and resubmit our manuscript entitled 'Chronically ill Patients’ Perspectives on Support Services and Activities of Patient Organizations' (Manuscript ID: JHPR-D-23-00131), which has been submitted to the Israel Journal of Health Policy Research.

We wish to thank the Reviewer and editor for their investment in this submission and the instructive remarks.

I have attached the revision of the manuscript in two files- one marked with tracked changes, and the other as a final version. Alongside, a detailed response letter addressing the feedback. In alignment with their insights, we have undertaken revisions to both clarify and enhance the text's quality.

We hope this manuscript revision satisfies the concerns raised by the Editor and Reviewer. Following are our detailed responses to each comment.

Sincerely,

Avi Zigdon on behalf of all authors

**Comments from Reviewer**

Reviewer #2:

This manuscript can be an important addition to IJHPR's publications related to patient-centered care.

1. I would like to see some re-writing in the Discussion and Conclusions.

**Authors' Response:** Thank you for your comment. The discussion and conclusions have been rewritten.

2. The last sentence in the manuscript reads, "This study tested the POQ on the general population of chronically ill patients, and further research is needed to test the POQ in different patient groups." I hate to see a paper in a policy journal end with the idea that more research is necessary. It makes much more sense to me that an idea such as that should come up in the Limitations, and, if the authors feel it is even necessary to restate that point, the conclusion should begin more like, "Despite the desirability of further research on x,y,z, based on the findings of the present study we can make the following firm recommendations." Even that doesn't need to be stated - it can just be in the mindset of the authors as they make a set of firm conclusions/recommendations.

**Authors' Response:** Thank you for your comment. The correction was made. This comment was corrected in the previous round. After the comments and additions to the manuscript were corrected in the first round, the sentence in question became obsolete and was removed from the conclusions section (page 22).

3. As a non-Israeli, I don't know enough about the interaction of the MoH and the four HMOs with POs, but I suspect it is relatively weak. The authors could do patients and patient organizations a service by specifying how the interactions could be strengthened. I think it requires the MoH and the HMOs to reach out to the patient organizations (note I still prefer to eliminate acronyms). In the instance of some clinical conditions, the MoH and the HMOs may need to help form an appropriate patient organization.

**Authors' Response:** Thank you, all acronyms have been eliminated, with the exception of recognized statistical terms.

The relationship between patient organizations and Health Maintenance Organizations is not applicable within the Israeli healthcare system. In Israel, the Ministry of Health serves as both the regulator and a provider of health services, while the four Health Maintenance Organizations act as the primary providers of healthcare services to the country’s citizens. These Health Maintenance Organizations can be viewed as the central arms of healthcare service delivery. However, competition exists among the Health Maintenance Organizations for "health consumers," meaning that a partnership between a single Health Maintenance Organizations and a patient organization could lead to inequality. To address this potential disparity, we recommend the establishment of a patient council within the Ministry of Health that would represent patients' interests across all four health funds. This council would ensure equitable representation and prevent any single Health Maintenance Organizations from gaining an unfair advantage. We have also refined the characteristics necessary for this patient council to effectively serve its purpose. We clarified and specified the ways in which we think the interactions between the Ministry of Health and Health Maintenance Organizations to patient organizations can be improved for the betterment of patients and POs alike (page 20 and page 22).
